# Potential Inhibitory Influence of miRNA 210 on Regulatory T Cells during Epicutaneous Chemical Sensitization

**DOI:** 10.3390/genes8010009

**Published:** 2016-12-27

**Authors:** Carrie Mae Long, Ewa Lukomska, Nikki B. Marshall, Ajay Nayak, Stacey E. Anderson

**Affiliations:** 1 Immunology and Microbial Pathogenesis Graduate Program, West Virginia University, Morgantown, WV 26505, USA; clong14@mix.wvu.edu; 2 Centers for Disease Control and Prevention, National Institute for Occupational Safety and Health, Allergy and Clinical Immunology Branch, Morgantown, WV 26505, USA; uvm3@cdc.gov (E.L.); nikki.marshall@inovio.com (N.B.M.); ajay.nayak@jefferson.edu (A.N.)

**Keywords:** microRNA, regulatory T cell, immunotoxicology, toluene diisocyanate, isocyanate, miR-210

## Abstract

Toluene diisocyanate (TDI) is a potent low molecular weight chemical sensitizer and a leading cause of chemical-induced occupational asthma. The regulatory potential of microRNAs (miRNAs) has been recognized in a variety of disease states, including allergic disease; however, the roles of miRNAs in chemical sensitization are largely unknown. In a previous work, increased expression of multiple miRNAs during TDI sensitization was observed and several putative mRNA targets identified for these miRNAs were directly related to regulatory T-cell (T_reg_) differentiation and function including Foxp3 and Runx3. In this work, we show that miR-210 expression is increased in the mouse draining lymph node (dLN) and T_reg_ subsets following dermal TDI sensitization. Alterations in dLN mRNA and protein expression of T_reg_ related genes/putative miR-210 targets (foxp3, runx3, ctla4, and cd25) were observed at multiple time points following TDI exposure and in ex vivo systems. A T_reg_ suppression assay, including a miR-210 mimic, was utilized to investigate the suppressive ability of T_regs_. Cells derived from TDI sensitized mice treated with miR-210 mimic had less expression of miR-210 compared to the acetone control suggesting other factors, such as additional miRNAs, might be involved in the regulation of the functional capabilities of these cells. These novel findings indicate that miR-210 may have an inhibitory role in T_reg_ function during TDI sensitization. Because the functional roles of miRNAs have not been previously elucidated in a model of chemical sensitization, these data contribute to the understanding of the potential immunologic mechanisms of chemical induced allergic disease.

## 1. Introduction

Occupational allergic disease is a significant health burden. A variety of diseases can be caused by workplace chemical exposures, including asthma, conjunctivitis, dermatitis, rhinitis, and urticaria [1]. Diisocyanates are a group of highly reactive chemicals characterized by the presence of double isocyanate functional groups; many of these chemicals are potent sensitizers and major causative agents of occupational allergic disease [2,3,4]. Toluene diisocyanate (TDI) is frequently utilized in the automobile industry and in the manufacture of polyurethane foams, paints, and coatings [5,6,7,8]. TDI is widely used throughout the U.S. and around the globe; the U.S. Environmental Protection Agency reports that U.S. production and importation of 2–4 and 2–6 TDI isomers rose above one billion pounds in 2006 [6]. While TDI has generally been classified as a T-helper type 2 (Th2) sensitizer, the immune response following exposure is more accurately characterized as a mixed Th1/Th2 response in both rodents and humans [1,2,9]. The principal routes of human exposure to TDI are inhalation and dermal contact [6] and sensitization leading to allergic disease has been documented for both routes [7,10]. Once the induction threshold has been reached, skin sensitization is thought to be sufficient for subsequent allergic disease of the respiratory tract [7], illustrating the systemic nature of TDI sensitization. Although TDI-induced allergic disease is an extremely relevant occupational health concern the pathogenic mechanisms of diisocyanate-induced allergic disease are not fully understood [5]. The identification of novel mediators of allergic disease may be necessary to obtain a full and complete understanding of the disease. Because of the occupational significance of and lack of validated identification strategies for chemical respiratory sensitizers like TDI [4,11], it is necessary to investigate and identify functional pathways and mechanisms that are involved in TDI sensitization. Specific understanding of disease mechanism may have direct implications in risk assessment, hazard communication and guidance used in the selection of safe products, work place interventions and training programs to warn workers about potential risks, the identification of safer alternatives, and the selection of proper personal protective equipment (PPE).

An emerging class of epigenetic regulatory elements that have been the subject of recent scientific focus are microRNAs (miRNAs). These molecules are single stranded, noncoding RNA molecules that are approximately 19–23 nucleotides long [12]. miRNAs exhibit functional significance though posttranscriptional gene regulation due to their ability to bind to the target mRNA and destabilize and inhibit protein translation when in complex with Argonaute proteins and the RNA-induced silencing complex (RISC). The seed sequence of a mature miRNA interacts with the 3′ untranslated region of target mRNA and leads to the translational repression or degradation of the target mRNA, influencing gene expression. Recently, it has been shown that miRNAs play a major role in a variety of immune responses [13,14,15,16]. These regulatory factors have not been functionally investigated in the context of chemical sensitization, with the exception of recent work by our group that profiled the general expression kinetics of miRNAs involved in TDI sensitization [1] and research profiling miRNA expression in both human and murine diphenylcyclopropenone-induced dermatitis [17].

The identification of increased expression of several miRNAs in the draining lymph nodes (dLN) of mice during epicutaneous TDI sensitization prompted further investigation into the potential functional roles of these molecules in the allergic response. Target analysis for upregulated miRNAs (miR-210, -31, and -155) revealed several putative and confirmed regulatory T cell (T_reg_)-related targets including *foxp3* and *runx3* in murine and human genomes [1]. These transcription factors and the signaling molecules CD25 and CTLA4 are integral to T_reg_ differentiation and function. The expression of these molecules allows T_reg_ to differentiate in response to allergens and exert immunoregulatory functions, dampening inappropriate inflammatory and adaptive immune responses. In addition, miR-31 and -155 have been implicated as regulators of T_reg_ in a variety of contexts [18,19]. A role for T_regs_ has been suggested in models of chemical-induced contact hypersensitivity [20,21] and, in a recent manuscript, Long et al. demonstrated the increased expression and functional capability of T_regs_ during TDI sensitization [21]. While the collection of data regarding roles for T_regs_ and miRNAs in chemical allergy is growing, it is still limited. Recently published data suggests an important role for T_regs_ in dermal TDI sensitization, yet the interaction between these cells and selected miRNAs has not been investigated. While miR-210 is well characterized in the hypoxia response, its specific role in allergic disease has not yet been defined. In the present study, we utilized a murine model of epicutaneous TDI sensitization in order to elucidate the expression kinetics and role of miR-210 and its putative mRNA targets in a murine model of epicutaneous TDI sensitization, specifically in relation to the T_reg_ subset.

## 2. Materials and Methods

### 2.1. Mice

Female BALB/c mice (6–8 weeks of age) were obtained from Taconic (Germantown, NY, USA), acclimated for 5 days, and then randomly assigned to treatment group; homogenous weight distribution was ensured across treatment groups. Mice were housed in ventilated plastic shoebox cages with hardwood chip bedding at a maximum of five animals per cage. A NIH-31 modified 6% irradiated rodent diet (Harlan Teklad, Frederick, MD, USA) and tap water were administered ad libitum. Housing facilities were maintained at 68–72°F and 36%–57% relative humidity, and a 12 h light–dark cycle was maintained. All animal experiments were performed in the Association for Assessment and Accreditation of Laboratory Animal Care (AAALAC) accredited National Institute for Occupational Safety and Health (NIOSH) animal facility in accordance with an Institutional Animal Care and Use Committee-approved protocol (protocol number 15-SA-M-004, date of approval 1 August 2015).

### 2.2. TDI Sensitization Model

Toluene 2,4-diisocyanate (TDI, CAS# 584-84-9) was obtained from Sigma-Aldrich (Milwaukee, WI, USA). Animals were exposed to a single dose of 0%, 0.5%, and 4% TDI (*v*/*v*) on the dorsal surface of each ear (25 µL per ear). The chosen TDI concentrations (0.5% and 4% *v*/*v*) and dosing regimen was previously shown to induce sensitization [1,21] and 4% TDI (1000 μg TDI/cm^2^) was previously reported as the minimum single dose concentration of TDI that could induce maximum sensitization in the absence of systemic toxicity [1]. Acetone was selected as the vehicle control and has been historically utilized in our laboratory to evaluate chemical sensitization [21,22,23]. It is important to note that due to hydrolysis, slight variations in the concentration of TDI dosing solutions may have occurred between preparation and application. However, animals were exposed within 30 min of TDI preparation and no visualization of hydrolysis was observed subsequent to exposures.

### 2.3. Euthanasia, Tissue Collection, and Processing

Animals were weighed, euthanized via CO_2_ asphyxiation at time points ranging from 1 to 11 days post chemical exposure, and examined for gross pathology. Left and right auricular draining lymph nodes (dLNs; drain the site of chemical application) were collected in 4 mL sterile phosphate-buffered saline (PBS, pH 7.4) and manually dissociated using the frosted ends of two microscope slides. Cells were counted using a Cellometer (Nexcelom Bioscience, Lawrence, MA, USA) and size exclusion parameters (3.5 to 36 µm) with a combined acridine orange/propidium iodide solution to identify viable cells. For isolation of specific cellular subsets Stemcell magnetic isolation kits (Vancouver, BC, Canada) were utilized, including the T_reg_ isolation kit (CD4 negative and CD25 positive selection).

### 2.4. Ex Vivo miRNA Transfection Assays

Following dLN processing and counting, miR-31, -155, and -210 mimics (25 pmol in Lipofectamine transfection reagent, ThermoFisher, Waltham, MA, USA) were reverse transfected in flat-bottom 24-well plates for 24 h. Then, 1.5 × 10^6^ naïve or acetone-treated dLN cells/mL (in RPMI-1640 media) were added to plates followed by general T cell stimulation (2 µg/mL α-CD3, 1 µg/mL α-CD28 and incubated in fresh media at 37 °C and 5% CO_2_. Following a 72 h incubation, cells were washed with RPMI-1640 and RNA was isolated as described in the following section. RNA was assayed for intracellular miRNA expression to conform the intracellular uptake of miRNA mimics as well as T_reg_-related gene expression via RT-PCR.

### 2.5. RNA Isolation, Reverse Transcription, and RT-PCR

Total RNA was isolated from the dLN using the miRNeasy kit (Qiagen, Hilden, Germany) according to the manufacturer’s directions. A QiaCube (Qiagen) automated RNA isolation machine was utilized in conjunction with the specified RNA isolation kit. The concentration and purity of the RNA was determined using a ND-1000 spectrophotometer (Thermo Scientific Nanodrop, Wilmington, DE, USA). For gene and primary miRNA expression analysis, first strand cDNA synthesis was performed using a High-Capacity cDNA Synthesis Kit (Applied Biosystems, Carlsbad, CA, USA) according to the manufacturer’s recommendations. For mature miRNA, reverse transcription TaqMan MicroRNA Assays (looped-primer RT-PCR; Applied Biosystems) were utilized according to manufacturer’s recommendations (both multiplex and singleplex protocols were utilized).

For analysis of mRNA and primary miRNA expression, TaqMan Universal Fast master mix (Life Technologies, Carlsbad, CA, USA), cDNA, and mouse-specific mRNA primers (TaqMan Custom PCR Arrays, Carlsbad, CA, USA) were combined and PCR was performed according to the manufacturer’s protocol (TaqMan Gene Expression Analysis). For analysis of miRNA expression, TaqMan Universal 2× master mix, No AmpErase UNG (Life Technologies), cDNA, and mouse-specific miRNA primers (TaqMan Custom PCR Arrays) were combined and PCR was performed according to manufacturer protocol (TaqMan miRNA Assays both Single- and Multi-Plex). Primers used include: *β-actin, cd25 (il2rα), ctla4, foxp3, mature miR-210, -31, -155, primary miR-210, runx3*, and *sno234*. MicroAmp Fast Optical 96-well reaction plates were analyzed on an Applied Biosystems 7500 Fast Real Time PCR system using cycling conditions as specified by the manufacturer. *β-actin* (mRNA and primary miRNA) and snoRNA234 (miRNA) were used as the endogenous reference control gene as expression was determined to be stable following chemical exposure (data not shown). RT-PCR data were collected and represented as relative fold change over vehicle control, calculated by the following formula: 2^−ΔΔCt^ = ΔCt_Sample_ − ΔCt_Control_. ΔCt = Ct_Target_ − Ct_β-ACTIN_, where Ct = cycle threshold as defined by manufacturer.

### 2.6. Flow Cytometric Analysis and T_reg_ Phenotyping

Single cell suspensions were prepared from tissues and a minimum of 150,000 dLN cells were aliquoted into 96-well U-bottom plates and washed in fluorescence-activated cell sorting (FACS) staining buffer (PBS + 1% bovine serum albumin + 0.1% sodium azide). Cells were resuspended in staining buffer containing anti-mouse CD16/32 antibody (clone 2.4G2; BD Biosciences, San Jose, CA, USA) for blocking of F_c_ receptors to minimize nonspecific binding. Cells were resuspended in staining buffer containing a cocktail of fluorochrome-conjugated antibodies specific for cell surface antigens including: CD3 (500A2, V500, BD Biosciences, Franklin Lanes, NJ, USA), CD4 (RM4-5, AF700, BD), CD8a (53-6.7, AF488, BioLegend, San Diego, CA, USA), CD25 (PC61, APC Cy7, BioLegend), CD45 (30-F11, PE, BD). Following surface staining, cells were washed in staining buffer and fixed using the Foxp3 fixation buffer set (eBioscience, San Diego, CA, USA). After overnight incubation in staining buffer, cells were permeablilized using the Foxp3 fixation buffer set (eBioscience) and re-suspended in permeabilization buffer containing a cocktail of fluorochrome-conjugated antibodies specific for intracellular antigens including: RUNX3 (R3-5G4, PE, BD), Foxp3 (FLK-16s, eF450 and APC, eBioscience). Following staining, cells were re-suspended in staining buffer and analyzed on an LSR II flow cytometer using FacsDiva software (BD Biosciences). Data analysis was performed with FlowJo 10.0 software (TreeStar Inc., Ashland, OR, USA). A minimum of 10,000 events were captured for each sample. Leukocytes were first identified by their expression of CD45. The T_reg_ subset was further identified as CD3^+^CD4^+^CD8^−^CD25^+^Foxp3^+^. Numerical population values were calculated by applying subset frequencies to the initial cell count obtained following lymph node homogenization. Compensation controls were performed using single stained cellular suspensions and OneComp beads (eBioscience, San Diego, CA, USA) and fluorescence minus one (FMO) staining controls were included to help set gating boundaries.

### 2.7. T_reg_ Suppression Assay

The suppressive ability of T_regs_ was analyzed using an ex vivo T_reg_ suppression assay as described by Long et al. [21] with modifications. This assay evaluates the ability of naïve, conventional dLN-derived T cells (T_cons_) to proliferate in the presence of varying numbers of T_regs_ isolated from acetone- or TDI-exposed mice. Mice were exposed to acetone (*n* = 7–11) or TDI (4%) (*n* = 4–5) as previously described and following sacrifice at 7 days (peak of the expansion) post TDI exposure the dLN and spleens were removed. T_regs_ (CD4^+^CD25^+^) and T_cons_ (CD4^+^CD25^−^) were isolated from the lymph nodes and CD4^−^ accessory cells were isolated from naïve spleens using CD4 negative and CD25 positive selection-based magnetic separation kits (Stemcell, Vancouver, BC, USA). Average T_reg_ purity is as follows for 7 days. Acetone: 96.5% ± 0.8% of CD3^+^CD4^+^ cells. 4% TDI: 97.35% ± 0.25% of CD3^+^CD4^+^ cells. Following isolation from naïve mouse dLNs, T_cons_ were labeled with 2 µM carboxyfluorescein succinimidyl ester (CFSE). miR-210 mirVana mimic (2 pmol; ThermoFisher) in Lipofectamine RNAiMAX (ThermoFisher) or Lipofectamine only control (LO) were added to a 96-well U-bottom plate in order to reverse transfect cells. T_cons_ and T_regs_ were cultured in a 96-well U-bottom plate with anti-CD3 (0.2 µg/mL; BD Biosciences) and accessory cells (naïve CD4^−^ splenocytes treated with mitomycin C) at a variety of T_con_:T_reg_ ratios (1:1, 2:1, 4:1, and 8:1). Additional controls included stimulated T_cons_ only to assess baseline proliferation, T_regs_ only, accessory cells only, and T_cons_ only with no stimulation nor accessory cells. Cells from each treatment group were pooled and added to triplicate wells of the culture plate. Seventy-two hours following plating, cells were stained with anti-CD4 and Live/Dead Violet (Life Technologies). T_cons_ were defined as CD4^+^CFSE^+^ cells and suppression was measured based on changes in the frequency of dividing CFSE^+^ cells based on the dilution of CFSE. T_regs_ were analyzed for purity based on their expression of CD3, CD4, and Foxp3 as determined by flow cytometric analysis as previously described.

### 2.8. Statistical Analysis

**Figure 4:**
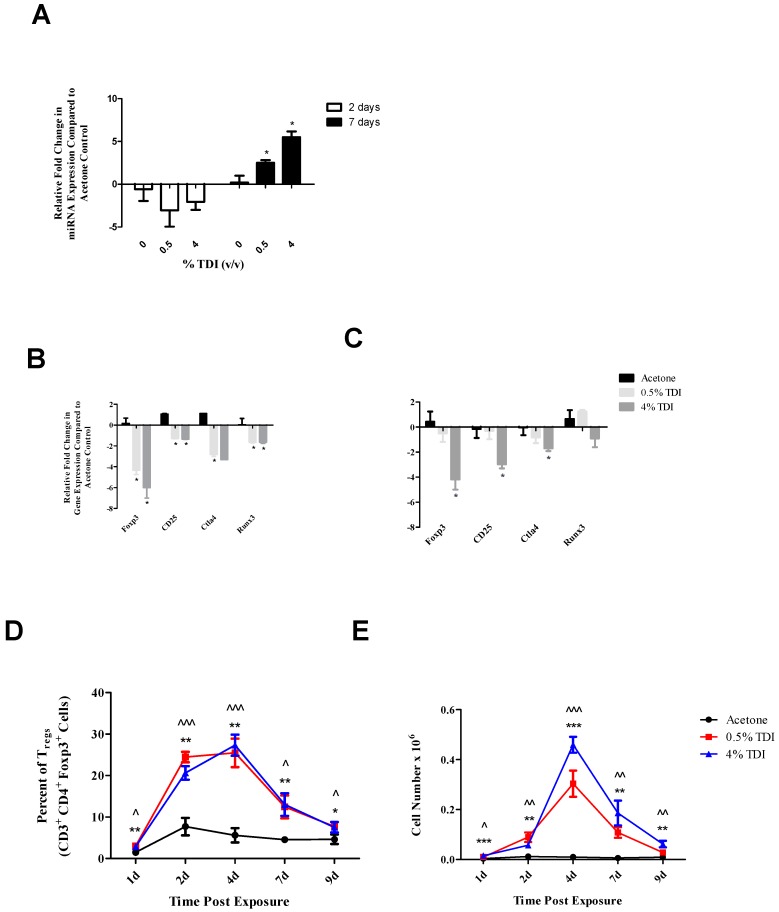
Mature miRNA and Runx3 expression increases in T_regs_ during TDI sensitization. RT-PCR analysis of miR-210 (**A**) and T_reg_-related gene expression in the dLN T_reg_ subset at two (**B**) and seven days (**C**) post TDI exposure. T_regs_ were isolated as CD4^+^CD25^+^ cells and cellular purity was assessed via flow cytometric staining for CD3^+^CD4^+^Foxp3^+^ events (2 days: % T_regs_ of all CD4^+^ events, mean, *n* = 4–5 per group: Acetone: 83.8% ± 1.6%, 0.5% TDI: 67.1% ± 4.2%, and 4% TDI: 72.3% ± 1.9%; 7 days: % T_regs_ of all CD4^+^ events: Acetone: 91%, 0.5% TDI: 88.4%, and 4% TDI: 86.7%). Flow cytometric analysis of dLN Runx3^+^ T_reg_ frequency (**D**) and number (**E**) following TDI sensitization. Bars represent mean relative fold change (±SE) of 4–5 mice per group. Significance is indicated by * *p* ≤ 0.05 for (**A**–**C**) and *p* ≤ 0.05 (*), *p* ≤ 0.01 (**), *p* ≤ 0.001 (***), and *p* ≤ 0.0001 (****) for 4% TDI or *p* ≤ 0.05 (^), *p* ≤ 0.01 (^^), *p* ≤ 0.001 (^^^), and *p* ≤ 0.0001 (^^^^) for 0.5% TDI compared to vehicle control (**D**,**E**).

Statistical analyses were generated using GraphPad Prism version 5.0 (San Diego, CA, USA). Data were analyzed by a Student *t*-test comparing groups as indicated in the figure legends. Figure 4C was analyzed by analysis of variance using PROC MIXED. In some cases, data were transformed using the natural log to meet the assumptions of the analysis. Significant interactions were explored utilizing the “slice” option in PROC MIXED and pairwise differences were assessed using a Fisher’s Least Significant Difference Test. All differences were considered significant at *p* < 0.05; representative significance symbols varied by figure, as indicated in the legend.

## 3. Results

### 3.1. Examination of miR-210 Expression during TDI Sensitization

**Figure 1:**
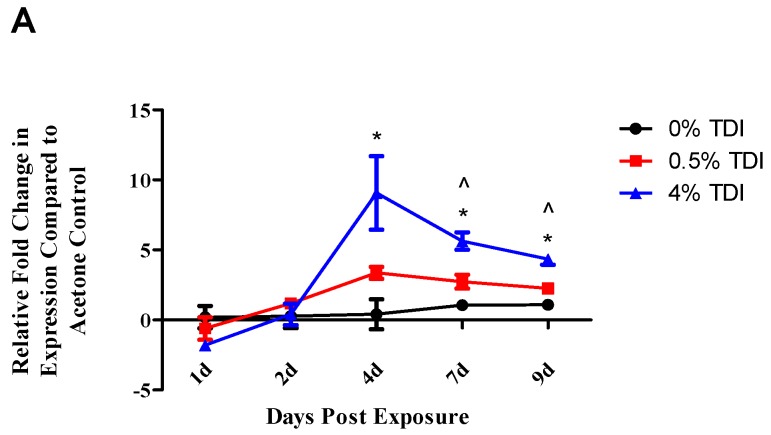

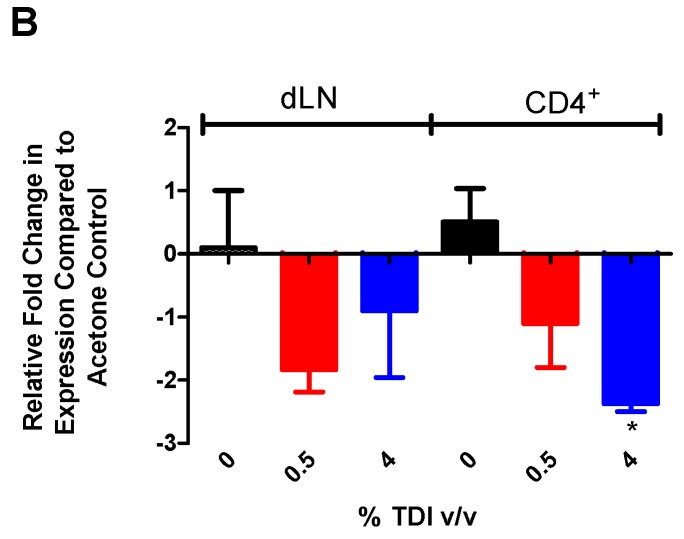
Mature and primary miR-210 expression increases in the draining lymph node (dLN) and CD4^+^ subsets following toluene diisocyanate (TDI) sensitization: RT-PCR analysis of mature miR-210 expression in the dLN at various time points post TDI exposure (**A**); and primary miR-210 expression in the whole dLN and dLN CD4^+^ subset four days post TDI exposure (**B**). Cellular purity was assessed via flow cytometric staining for CD4^+^ Cells (% CD4^+^ of all cells; Acetone: 89.6% ± 2.2%, 0.5% TDI: 91.5% ± 0.3%, and 4% TDI: 90.4% ± 0.3%). Bars represent mean relative fold change (± standard error, SE) of 3–5 mice per group. Statistical significance is represented by ^ (0.5% TDI) and * (4% TDI) (*p* < 0.05) compared to vehicle control.

The kinetics of mature miR-210 expression were investigated via RT-PCR in the dLN following 0.5% and 4% TDI exposure. As previously reported [1], dLN miR-210 increased at various time points during 0.5% and 4% TDI sensitization, including four (4%), seven, and nine (0.5% and 4%) days post single exposure, with expression appearing to peak at four days post 4% TDI exposure (Figure 1A). Primary miRNA (pri-miRNA) transcripts represent the early precursor stage of mature miRNA structure prior to modification and cleavage by Drosha and Dicer. Pri-miR-210 levels were analyzed in the dLN at four days post TDI exposure, which represents the relative peak of dLN miR-210 expression. Although expression of pri-miR-210 was detected in the dLN, no significant alterations were observed. Alternatively, pri-miR-210 expression significantly decreased in the CD4^+^ subset during 4% TDI sensitization (Figure 1B).

### 3.2. Ex Vivo miR-210 Mimic Transfection Reveals Potential Inhibitory Effect of miR-210 on T_reg_-Related Genes

**Figure 2:**
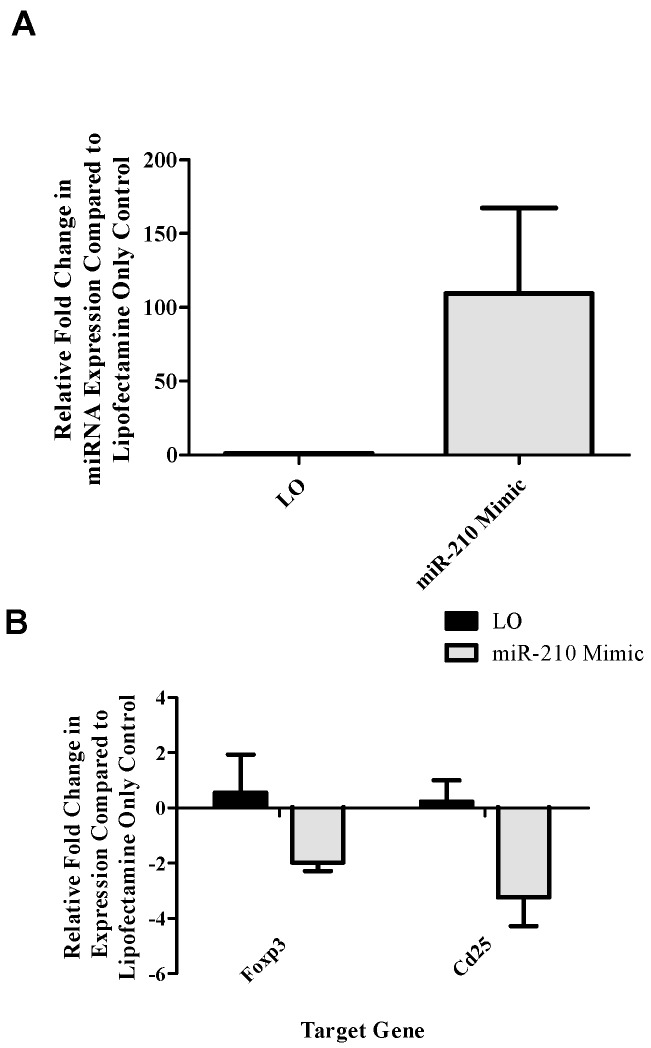
Ex vivo miR-210 mimic augmentation reveals potential inhibitory effect of miR-210 on regulatory T cell (T_reg_)-related genes in dLN cells. (**A**) Intracellular miR-210 was quantified via RT-PCR in ex vivo stimulated samples 72 h following transfection with lipofectamine only control (LO) or miR-210 mimic. (**B**) *foxp3* and *cd25* (*il2rα*) were investigated as potential miRNA targets via RT-PCR. Bars represent mean relative fold change (± SE) of 3 replicates per group.

Due to the putative link between miR-210 and T_reg_-related targets in both human and mouse-based studies and target algorithms [1], an ex vivo transfection and stimulation assay was set up to directly examine miR-210 and T_reg_-related gene expression. Analysis of T_reg_-related gene expression induced by the addition of excess levels of miR-210 was examined. Although not statistically significant, Figure 2A reveals increased miR-210 expression in cells treated with miR-210 mimic (101 fold) as compared to lipofectamine only (LO) control (1.2 fold). Analysis of T_reg_-related genes revealed decreased trends of expression of T_reg_-related genes following miR-210 mimic transfection compared to the LO control, including *foxp3* (~−2.0 fold compared to 0.6 fold) and *cd25* ((~−3.2 fold compared to 0.2 fold) (Figure 2B)). No apparent changes in *runx3* or *ctla4* were observed (data not shown).

### 3.3. In Vivo dLN Target Expression Reveals Decreased Expression of Several Key T_reg_-Related Genes

**Figure 3:**
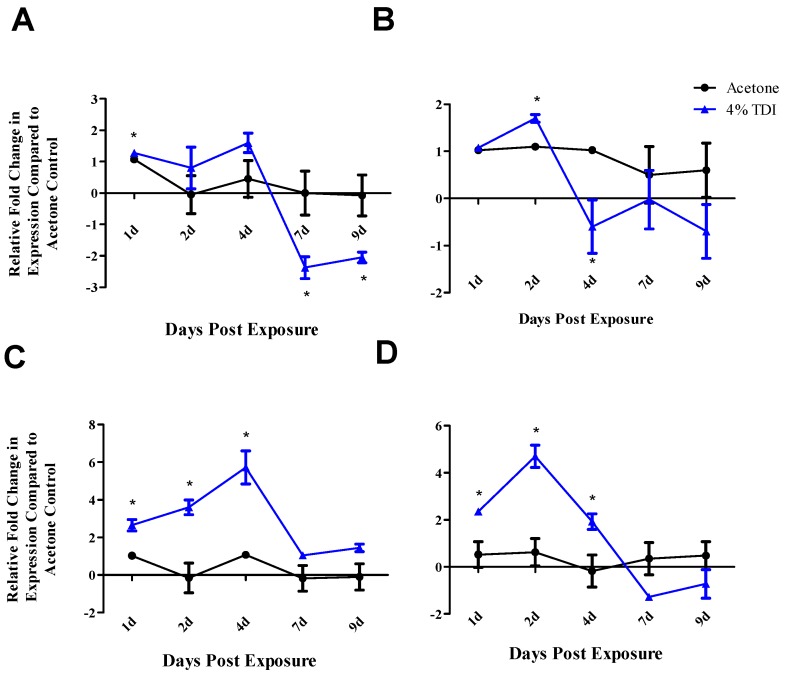
In vivo dLN mRNA expression of potential miR-210 targets reveals altered expression during TDI sensitization. RT-PCR analysis of: *foxp3* (**A**); *runx3* (**B**); *ctla4* (**C**); and *cd25* (**D**) expression in the whole dLN at various time points post 0% and 4% TDI exposure. Bars represent mean relative fold change (± SE) of five mice per group. Statistical significance is represented by * (*p* < 0.05) compared to the vehicle control.

The expression of potential miR-210 targets and key T_reg_ genes potentially affected by miR-210 was investigated in the whole dLN at various time points following 4% TDI exposure. Similar to the findings of the ex vivo study and previously reported findings, dLN target/key player mRNA expression was significantly decreased at four (*runx3*), seven (*foxp3*), and nine (*foxp3*) days post 4% TDI exposure (Figure 3A,B). *Foxp3* expression was decreased by approximately threefold in comparison to the acetone control at seven and nine days post TDI exposure (Figure 3A). Early statistically significant increases in *foxp3* (one day) and *runx3* (two day) were also observed but only for a single time point and not determined to be of biological significance. dLN *ctla4* and *cd25* expression significantly increased with peak expression at four and two days post 4% TDI exposure, respectively. However, these increases in expression were not maintained at later time points (Figure 3C,D).

### 3.4. T_reg_-Specific miRNA and Gene Expression

Due to the suspected influence of miR-210 on T_regs_, the levels of this miRNA were examined in T_regs_ (CD4^+^CD25^+^) from the dLN of mice treated with 0%, 0.5%, and 4% TDI at two and seven days post exposure. These time points were selected to reflect early and peak miR-210 and T_reg_ responses in the dLN (Figure 1A and [1]). While no statistically significant changes were observed following two days of exposure, miR-210 levels were significantly increased in T_regs_ during 0.5% and 4% TDI sensitization in a dose responsive fashion at seven days post exposure (Figure 4A). Expression of T_reg_-related genes was also examined in isolated T_regs_ following TDI exposure. Significant decreases in *foxp3*, *ctla4*, *runx3*, and *cd25* were observed at both concentrations (except for *ctla4* at 4%) at two days post exposure (Figure 4B). Similarly, significant decreases in *foxp3*, *ctla4*, and *cd25* mRNA were observed in T_regs_ from 4% TDI-exposed mice at seven days post 4% TDI exposure (Figure 4C), further indicating that these genes may be influenced by miR-210 (Figure 1A). T_reg_-specific expression of *runx3* was assayed in dLN T_regs_ during TDI sensitization. In contrast to the mRNA levels, increases in frequency and numbers of Runx3^+^ T_regs_ at all time points were observed following 0.5% and 4% TDI exposure (Figure 4D,E).

### 3.5. The Ex Vivo Suppressive Capability of T_regs_ Is Influenced by miR-210 Levels

**Figure 5:**
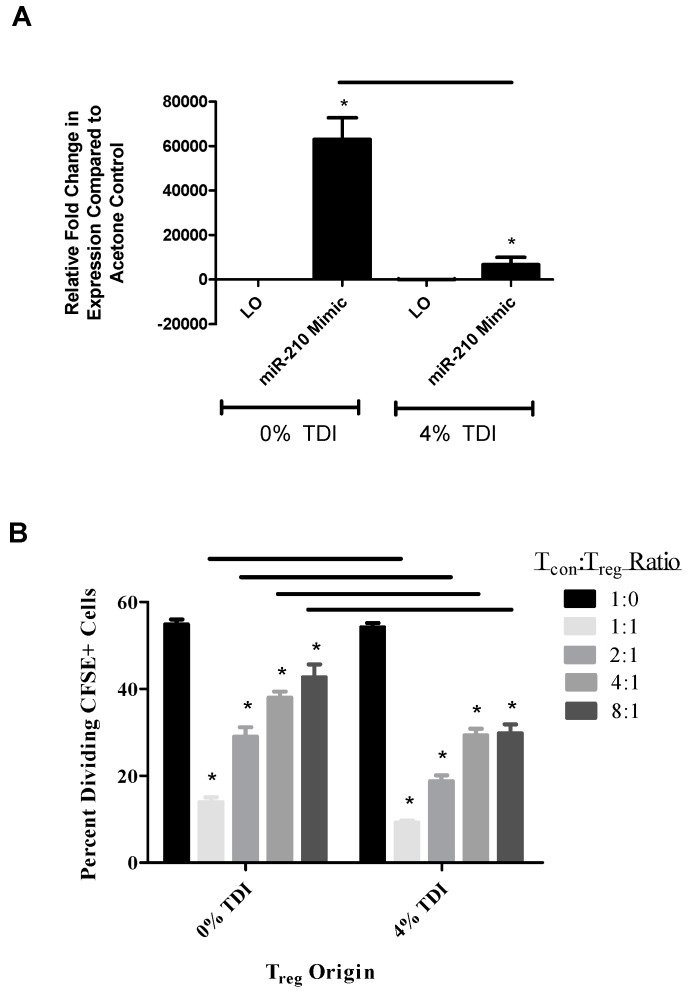
Increased T_reg_ suppression is associated with decreased miR-210 levels. A CFSE-based T_reg_ suppression assay was performed with T_regs_ from mice treated with acetone or TDI (seven days post exposure) and the addition of miR-210 mimic. RT-PCR analysis of intracellular miR-210 expression for acetone or TDI treated mice following addition of miR-210 mimic or Lipofectamine only (LO) control (**A**). Statistical significance is represented by horizontal lines comparing indicated groups and asterisks (compared to LO control) (*p* < 0.05). Functional capacity of T_regs_ based on percent dividing CFSE^+^ naive conventional T cells (T_con_) at indicated ratios following addition of miR-210 mimic (**B**). *p* values are represented by * (*p* < 0.05; comparison of each treatment group to 1:0 ratio from the same chemical treatment group) or horizontal bars (comparison of identical ratios between different mimic treatment groups). Bars represent mean relative fold change (± SE) of three replicates per group.

In order to examine the suspected inhibitory role of miR-210 on T_regs_ during TDI sensitization, the functional capabilities of T_regs_ were tested in an ex vivo suppression assay. miR-210 mimic was transfected in selected wells utilizing a lipid-based transfection strategy and intracellular miR-210 mimic uptake was confirmed in these wells at the time of cellular harvest (Figure 5A). Interestingly, following addition of the miR-210 mimic, miRNA-210 levels were almost 10-fold higher in wells containing acetone T_regs_ compared to those with TDI T_regs_ (Figure 5A). No differences were noted between miR-210 levels among the acetone and TDI groups treated with LO (Figure 5A). Significant baseline T_reg_ suppressive ability was observed in wells with Lipofectamine only and acetone-derived T_regs_ as well as 4% TDI-derived T_regs_ (data not shown). This was not altered significantly upon the addition of miR-210 mimic with the exception of the 1:1 ratio in wells with 4% TDI-derived T_regs_ (data not shown). As previously evidenced [21], TDI-derived T_regs_ exhibited greater suppressive capability than acetone-derived T_regs_ in mimic-treated wells for the 1:1, 2:1, 4:1, and 8:1 ratios (Figure 5B). This increased function was observed in the presence of lower miR-210 levels suggesting that TDI may induce additional factors that might influence the functional capabilities of the T_regs_.

### 3.6. Further Investigation of miR-31 and -155 Suggest Additional Regulation of the TDI Sensitization Response, Potentially Impacting miR-210 and T_regs_

**Figure 6:**
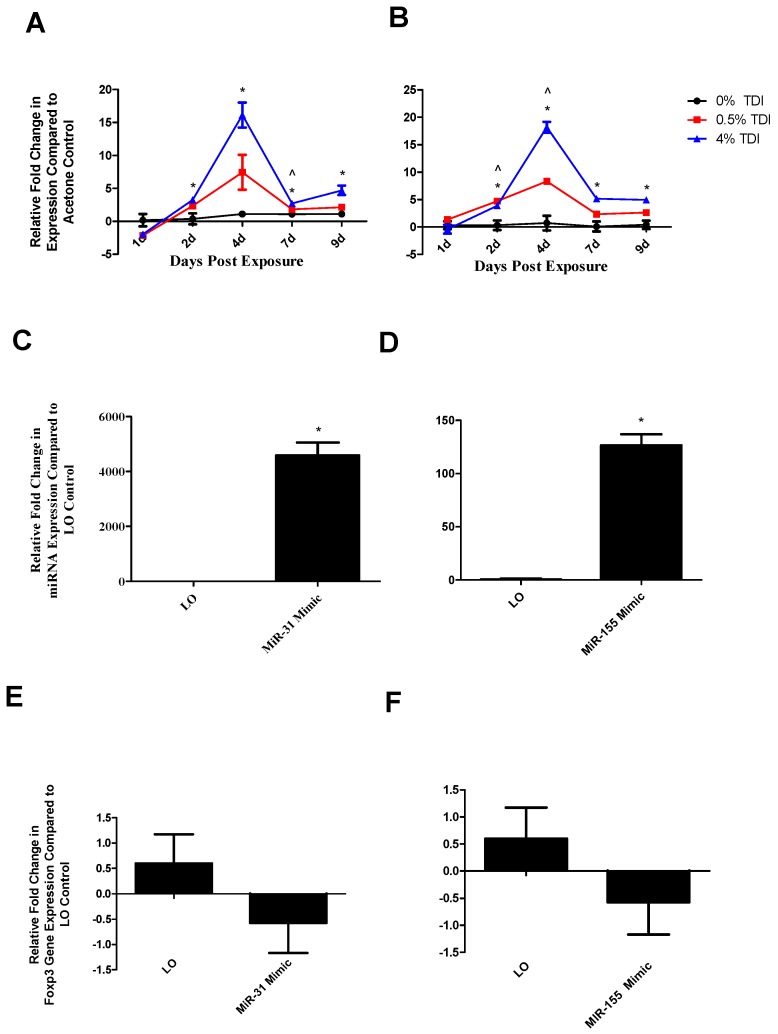
In vivo dLN miR-31 and -155 levels increase during TDI sensitization. Whole dLN miR-31 (**A**) and -155 (**B**) levels were quantified via RT-PCR at various time points post TDI sensitization. Statistical significance is represented by ^ (0.5% TDI) and * (4% TDI) compared to vehicle control (*p* < 0.05). Intracellular miR-31 (**C**), -155 (**D**) was quantified via RT-PCR in ex vivo stimulated samples (with miR-31 or -155 mimic addition, respectively) after 72 h. (**D**–**F**) *foxp3* expression was investigated in cultures following miR-31 (**D**) and -155 (**E**) mimic treatment. Statistical significance is represented by * when compared to lipofectamine only (LO) control (*p* < 0.05). (*n* = 3–5/group).

Evaluation of additional miRNAs was performed in an attempt to identify other potential factors that might influence miR-210 and T_reg_ function. miR-31 and -155 were selected since they have been identified to significantly increase in dLN expression earlier in TDI sensitization compared to miRNA-210 (Figure 6A,B). Ex vivo target analysis was performed utilizing additional miRNA mimics including miR-31 and -155 in order to further pursue the potential regulation of the T_reg_ subset by these miRNAs in addition to miR-210. Significantly increased intracellular levels of miR-31 (Figure 6C) and miR-155 (Figure 6D) were confirmed 72 h following transfection and stimulation. Although not statistically significant, apparent decreases in *foxp3* expression were observed following miR-31 (Figure 6E) and miR-155 (Figure 6F) mimic transfection. No changes in expression were observed for *runx3*, *ctla4*, or *cd25* following mimic addition (data not shown).

**Figure 7:**
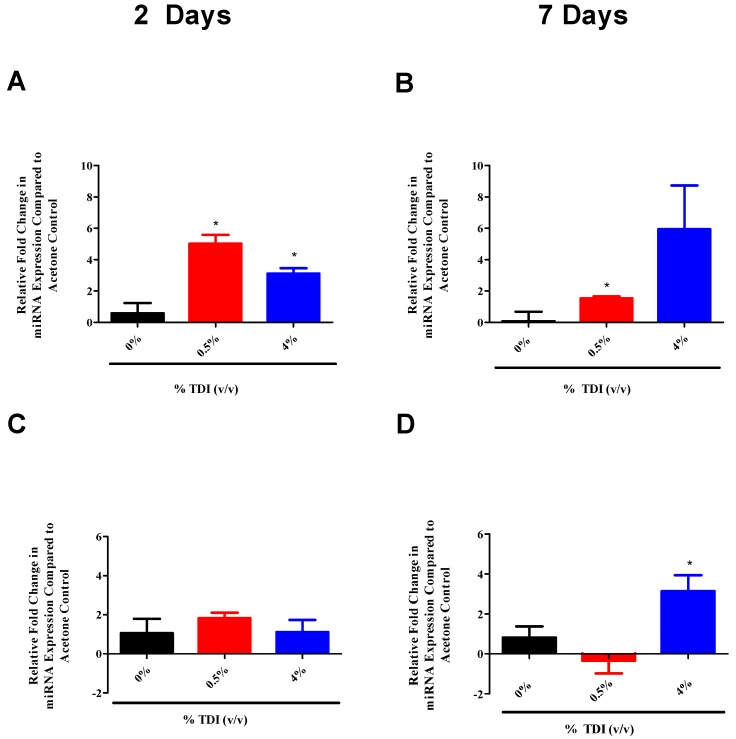
Mature miR-31 and -155 expression increases in T_regs_ during TDI sensitization. RT-PCR analysis of miR-31 (**A**,**B**) and -155 (**C**,**D**) expression in the dLN T_reg_ subset at two and seven days post TDI exposure. T_regs_ were isolated as CD4^+^CD25^+^ cells and cellular purity was assessed via flow cytometric staining for CD3^+^CD4^+^Foxp3^+^ events (2 days: % T_regs_ of all CD4^+^ events, mean, *n* = 4–5 per group: Acetone: 83.8% ± 1.6%, 0.5% TDI: 67.1% ± 4.2%, and 4% TDI: 72.3% ± 1.9%; 7 days: % T_regs_ of all CD4^+^ events, Acetone: 91%, 0.5% TDI: 88.4%, and 4% TDI: 86.7%). Bars represent mean relative fold change (± SE) of 4–5 mice per group. Statistical significance is represented by * (*p* < 0.05).

T_reg_-specific expression of miR-31 and miR-155 were also analyzed, revealing increased miR-31 levels two and seven days post 0.5% TDI exposure (Figure 7A,B) and increased miR-155 levels seven days post 4% TDI exposure (Figure 7D). Since miR-31 was identified to increase in expression earlier than miR-210 in the dLN and T_regs_, miR-210 expression was evaluated in dLN cells treated with miR-31 and -155 mimics. However, no changes in expression were observed. Similarly, expression of miR-31 and -155 was analyzed in cells treated with miR-210 mimic but no significant changes in expression were observed (data not shown).

## 4. Discussion

The occupational use of sensitizing chemicals such as TDI remains a significant public health concern. There are no validated hazard identification strategies for respiratory sensitizers like TDI and the complete immunologic mechanisms of sensitization have not been elucidated for these agents, hindering development of appropriate preventative assays. This justifies research pertaining to the identification of novel cellular subsets and epigenetic regulatory mechanisms such as miRNAs that may be involved in the respiratory chemical sensitization process. Following the identification of several upregulated dLN miRNAs during TDI sensitization, these molecules were investigated, specifically in relation to T_reg_ development and functionality. To our knowledge, this is the first work that functionally investigates miRNAs in a model of TDI-induced chemical sensitization.

miRNAs are powerful regulatory molecules which have been implicated in a number of immunologic states and conditions, including allergic disease [24,25,26]. Specifically, miR-155 has demonstrated a critical role in the development of antibody responses and germinal center function [27], miR-326 has been shown to regulate Th17 differentiation, exhibiting critical involvement in multiple sclerosis pathogenesis [28], and in vivo miR-126 inhibition reduces a house dust mite-induced asthmatic phenotype, demonstrating the importance of this miRNA in the regulation of Th2 responses and allergic asthma [24]. Vennegaard et al. described upregulation of several miRNAs, including miR-21, in skin biopsies from patients with allergic responses to diphenylcyclopropenone and in a murine model of dinitrofluorobenzene (DNFB) allergic contact dermatitis [17]. Additionally, previous work from our groups identified upregulation of several miRNAs, including miR-31, -155, and -210, in a murine model of epicutaneous TDI sensitization [1]. These ubiquitous signaling molecules are well-established mediators of many of signaling pathways in a number of cell types; however, their role in chemical sensitization is not well understood. For the work described in this manuscript, miR-210 was selected for additional investigation since it has been predicted and demonstrated to target T_reg_-related genes. In addition, its role in chemical sensitization and T_reg_ regulation has not yet been described.

The expression of miR-210 was quantified in a variety of tissues and cellular subsets in a murine model of TDI sensitization. Consistent with previous findings [1], increased expression of miR-210 in the dLN was also demonstrated in the present study during TDI sensitization (Figure 1A). In addition, increased expression of miR-210 was also identified in T_regs_ during TDI sensitization (Figure 4A). Since miRNA can be transported to cells via mechanisms such as exosomal transport, experiments to determine if cells are actively producing miR-210 following TDI exposure were conducted. The expression of pri-miRNA indicates gene level expression, presumably within the cell type tested. Pri-miR-210 was detected in the dLN and CD4^+^ subsets four days post TDI exposure (Figure 1B), indicating that miR-210 is being expressed in this tissue by CD4^+^ T cells. Interestingly, although they were detectable, pri-miR-210 levels significantly decreased in CD4^+^ T cells four days post 4% TDI exposure compared to equivalent cells in acetone-exposed mice, potentially indicating that the majority of mature miR-210 in the dLN is being produced by another cell type, is being transported from another tissue, or is being transcriptionally downregulated at this point, the peak of mature miR-210 levels in the dLN. While pri-miR-210 levels in T_regs_ were not investigated in the current study, previous studies have demonstrated miR-210 expression following the polarization of naïve T cells into T_regs_ [29].

Since T_regs_ have been implicated as regulators of TDI sensitization [21] and miR-210 expression has been shown to increase in T_regs_ following TDI sensitization (Figure 4A), an ex vivo target analysis system was designed in order to directly examine the effects of miR-210 on T_reg_-related genes. Selected genes identified as target of miR-210 included *foxp3*, the master transcription factor of the T_reg_ subset; and *runx3*, a transcription factor that signals upstream of *foxp3* by binding to this gene’s promoter [30]. Additionally, *cd25* and *ctla4* were investigated as important T_reg_-related genes as they are involved in IL-2 signaling and proliferation along with direct suppressive functions, respectively. This setup revealed a potential role for miR-210 in the downregulation of T_reg_-associated genes (*foxp3* and *cd25*; Figure 2B) following the addition of miR-210 mimic. In addition *foxp3* and *runx3* dLN expression decreased in the dLN at various time points following TDI exposure (Figure 3A,B) which is consistent with our previously reported findings [21]. The earlier decrease in *runx3* expression observed at four days post 4% TDI exposure may be reflective of the upstream signaling activity of this transcription factor in relation to *foxp3* (Figure 3B). While the findings for the ex vivo assay did not reach statistical significance, further support for T_reg_-associated genes as miRNA targets was provided by in vivo data. Interestingly, whole dLN expression of *ctla4* and *cd25* increased at one, two and four days post 4% TDI exposure (Figure 3C,D) which is likely a reflection of the activation of both T_regs_ and conventional T cells as elevated protein expression of these molecules is observed in T_regs_ at these time points [21] and would likely be increased in conventional T cells involved in TDI sensitization as well. In T_regs_, decreases in *foxp3*, *cd25* and *ctla4* were observed at two and seven days with *runx3* only being decreased at the earlier time point post TDI exposure (Figure 4B). These early changes in T_reg_ factors provide further support that additional factors might be involved in T_reg_ regulation since peak increases in miR-210 occur later than two days; accordingly, miR-31 was shown be increased at this time point. In contrast to the transcript, the expression kinetics of Runx3 increased following TDI exposure (Figure 4D). This expression pattern was similar to other T_reg_ proteins such as CD25 and Foxp3 (which are represented by the general T_reg_ population) and T_reg_-specific CTLA4 expression, which have previously been investigated during TDI sensitization [21]. The kinetics of T_regs_ bearing these molecules tended to peak at four days post TDI exposure with a relative decrease in both cellular frequency and number at seven days post TDI exposure [21]. In relation to miR-210 expression kinetics, this data may suggest that miR-210 has a regulatory role on the T_reg_ subset, as its expression wanes in concert with the general T_reg_ population as well as CTLA4^+^ and Runx3^+^ T_regs_. Additionally, miR-210 is a putative *runx3* target, suggesting a potential direct effect on this gene [1]. Collectively, this data is suggestive of T_reg_ regulation with visible effects on the expression of proteins beginning at Day 7 post TDI exposure. miR-210 expression remains elevated in the dLN throughout nine days post 0.5% and 4% TDI exposure (Figure 1A) and in T_regs_ at seven days post 0.5% and 4% TDI exposure (Figure 4A).

Due to the potential link between miR-210 and T_reg_-related gene expression, the functional capabilities of T_regs_ (acetone and TDI-derived) were examined in the presence and absence of miR-210 mimic. Interestingly, it appeared that miR-210 levels were lower in wells containing T_regs_ from TDI-treated mice and miR-210 mimic compared to wells with acetone-derived T_regs_ and miR-210 mimic (Figure 5A). The increased suppressive capability of TDI T_regs_ with miR-210 mimic (Figure 5B) may be a reflection of reduced miR-210 levels, as we hypothesize that miR-210 is inhibiting T_reg_ differentiation and/or function. This finding suggested that other regulatory factors including other miRNAs might be involved in the regulation of T_reg_ function. Complex interactions and interplay have often been reported for other miRNAs [31], therefore this concept was evaluated in the current study. Typically, direct miRNA–miRNA interactions are mediated by reverse complementary binding, resulting in the formation of duplexes [31]. Additionally, indirect miRNA–miRNA interaction may occur via target gene interaction; e.g., if a miRNA targets a gene that induces a different miRNA, this miRNA is being regulated by its own species.

miRNA-31 and-155 were further investigated for the potential to regulate the expression of miR-210 as they were identified to increase at early time points in TDI sensitization in the dLN (Figure 6A (miR-31) and B (miR-155)) and T_regs_ (Figure 7A (miR-31)). Similarly to miR-210, miR-31 and -155 were shown to potentially downregulate *foxp3* expression in this assay (Figure 6E,F). Although limitations in the assay sensitivities did not reflect significant changes, this may be reflective of a direct effect on *foxp3* or an indirect effect on this gene via other signaling pathways such as miR-210. These alterations are in accordance with recent findings pertaining to miRNA–mRNA interactions. miR-31 may indirectly target *foxp3*, leading to suppressed iT_reg_ development [19], accounting for the potential decreases in this gene evidenced following miR-31 mimic transfection. In addition, miR-31 increases earlier when more persistent decreases in *foxp3* were observed. Additionally, miR-155 expression appears to be controlled by *foxp3* in T_regs_ via binding to the intron within the DNA sequence encoding Bic, the precursor transcript of miR-155; accordingly, T_reg_ miR-155 levels have been shown to be highly responsive to *foxp3* levels [32]. This regulation may be interrupted by abnormally high levels of miR-155 in the mimic transfection system, resulting in decreased *foxp3* expression in these conditions via signaling feedback. This data suggests that miR-31 and -155 may be influencing the expression of miR-210 and/or T_regs_, possibly acting as early signaling mediators in the TDI sensitization response.

The lack of significance associated with the ex vivo experiments conducted in this work could be a reflection of experimental variability associated with similar assays and temporal discrepancies associated with signaling events. The ex vivo system displays several limitations, explaining the utilization of the in vivo TDI sensitization model in the target investigation as well. The T_reg_-related gene alterations that were observed as a consequence of miRNA mimic transfections in this system may indicate direct and/or indirect targeting by the miRNA. We propose that for the majority of miRNA–mRNA interactions investigated in our model, regulation is indirect, as few, if any, putative binding sites were identified for many of the potential targets and the corresponding miRNA. For example, miR-210 is predicted to target the 3′ UTR of *runx3* [1] and although we did not observe significant alterations in *runx3* expression following miR-210 mimic transfection in our ex vivo system, the in vivo expression kinetics of *runx3* suggest potential regulation. The ubiquitous nature of miRNAs and their involvement in various signaling processes accounts for their functional significance but can also cloud investigations into their mechanistic functions.

It is important to note that the increases in miRNA expression were not dependent on the irritant response, as dLN miR-210 levels significantly increased (Figure 4A) following the non-irritating [21] 0.5% TDI exposure. As 4% TDI exposure causes significant dermal irritation [21], ear miR-210 expression was analyzed at both non-irritant (0.5%) and irritant (1%, 2%, and 4%) TDI concentrations, revealing significant increases in dLN at both non-irritant and irritant doses (Figure 1A). In addition, other miRNAs including miR-22, -31, and -301a were also shown to increase significantly in expression regardless of the irritant status of the TDI dose (data not shown). This data prompts insight into the concept of the “two-signal” sensitization hypothesis which states that antigen delivery alone is insufficient for effective immunological priming but rather a second, innate signal is necessary to ensure the development of sensitization [33,34]. As noted in previous studies, the irritant response appears to be a prerequisite for strong sensitization responses in the case of dermal TDI sensitization [21]. Regardless, the expression of multiple miRNAs in the dLN appears to be due to the sensitization response alone and not significantly influenced by the irritant component of this response, which may be revealing as to their supposed functional roles in the sensitization response and may suggest potential utility as biomarkers of sensitization.

**Figure 8:**
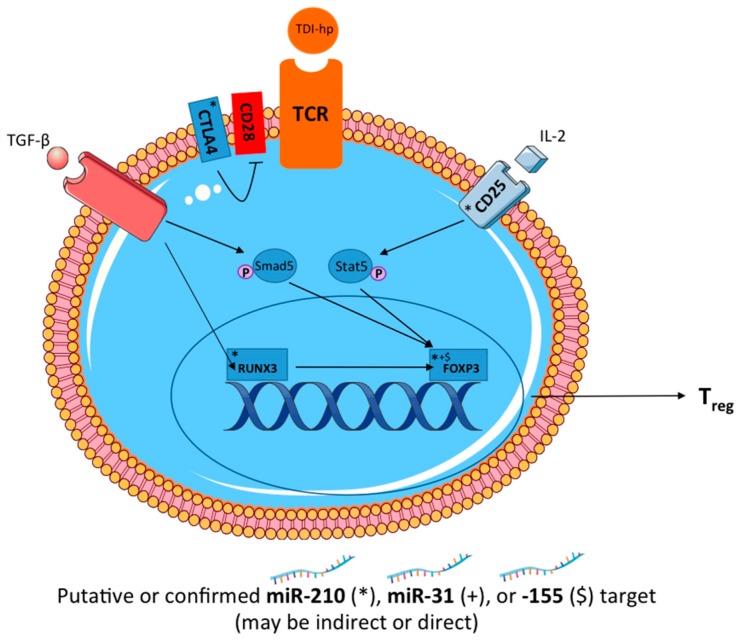
Proposed model of selected miRNA action on the regulatory T cell pathway. Integral components of the T_reg_ activation pathway are highlighted alongside putative direct targets or key players indirectly impacted by miRNA-210 (indicated by *), -31 (indicated by +), and -155 (indicated by $). Legend: CD28, Cluster of differentiation 28; CD25, IL-2 receptor alpha (in complex with IL-2Rβ and γc); CTLA4, Cytotoxic T-lymphocyte associated protein 4; Foxp3, Forkhead box P3; P, Phosphate group; Runx3, Runt related transcription factor 3; Smad5, Mothers against decapentaplegic homolog 5; Stat5, Signal transducer and activator of transcription 5, TCR, T cell receptor; TDI-hp, Toluene diisocyanate haptenated complex; TGF-β1, Transforming growth factor beta 1.

These studies reveal a potential role for miR-210 in a murine model of dermal TDI sensitization (Figure 8). Additionally, miR-31 and miR-155 were investigated for their regulatory potential in this response. The investigation of novel mediators of chemical-induced allergic disease is important for the overall understanding of the mechanisms involved in these responses. Therefore, this data may result in enhanced understanding of the mechanisms involved in chemical sensitization and could potentially aid in the development of hazard identification strategies for respiratory chemical sensitizers. In conclusion, we have demonstrated that miR-210 may negatively influence the differentiation and/or function of T_regs_ via direct targeting of *runx3* and/or indirect actions on other T_reg_-related genes. These findings suggest that these miRNAs may work in concert to affect the differentiation and function of T_regs_ as well as the expression and function of miR-210.
